# Long-term efficacy and safety of eladocagene exuparvovec in patients with AADC deficiency

**DOI:** 10.1016/j.ymthe.2021.11.005

**Published:** 2021-11-08

**Authors:** Chun-Hwei Tai, Ni-Chung Lee, Yin-Hsiu Chien, Barry J. Byrne, Shin-Ichi Muramatsu, Sheng-Hong Tseng, Wuh-Liang Hwu

**Affiliations:** 1 Department of Neurology, National Taiwan University Hospital and National Taiwan University College of Medicine, Taipei, Taiwan; 2 Department of Medical Genetics and Pediatrics, National Taiwan University Hospital and National Taiwan University College of Medicine, Taipei, Taiwan; 3 Powell Gene Therapy Center and Departments of Molecular Genetics and Microbiology and Pediatrics, University of Florida, Gainesville, FL, USA; 4 Division of Neurological Gene Therapy, Center for Innovation, Jichi Medical University, Shimotsuke, Japan; 5 Center for Gene & Cell Therapy, The Institute of Medical Science, The University of Tokyo, Tokyo, Japan; 6 Department of Surgery, National Taiwan University Hospital and National Taiwan University College of Medicine, Taipei, Taiwan

**Keywords:** aromatic L-amino acid decarboxylase deficiency, gene therapy, eladocagene exuparvovec, adeno-associated virus, putamen

## Abstract

Aromatic L-amino acid decarboxylase deficiency results in decreased neurotransmitter levels and severe motor dysfunction. Twenty-six patients without head control received bilateral intraputaminal infusions of a recombinant adeno-associated virus type 2 vector containing the human aromatic L-amino acid decarboxylase gene (eladocagene exuparvovec) and have completed 1-year evaluations. Rapid improvements in motor and cognitive function occurred within 12 months after gene therapy and were sustained during follow-up for >5 years. An increase in dopamine production was demonstrated by positron emission tomography and neurotransmitter analysis. Patient symptoms (mood, sweating, temperature, and oculogyric crises), patient growth, and patient caretaker quality of life improved. Although improvements were observed in all treated participants, younger age was associated with greater improvement. There were no treatment-associated brain injuries, and most adverse events were related to underlying disease. Post-surgery complications such as cerebrospinal fluid leakage were managed with standard of care. Most patients experienced mild to moderate dyskinesia that resolved in a few months. These observations suggest that eladocagene exuparvovec treatment for aromatic L-amino acid decarboxylase deficiency provides durable and meaningful benefits with a favorable safety profile.

## Graphical abstract

**Figure:**
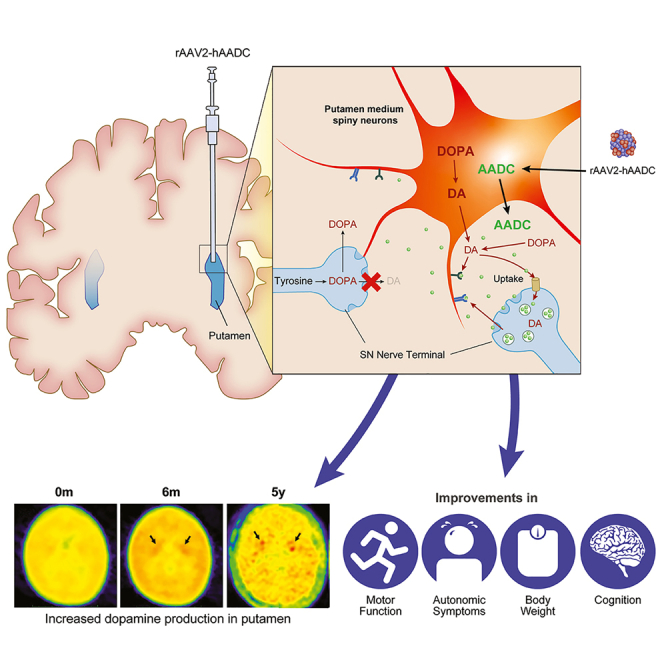

## Teaser

In this manuscript, corresponding authors and colleagues report the combined safety and efficacy results of a long-term follow-up from the three eladocagene exuparvovec gene therapy trials. These analyses provide important advancements in understanding the use of gene therapy in patients with AADC deficiency.

## Introduction

Aromatic L-amino acid decarboxylase (AADC) deficiency is a rare genetic neurological disorder arising from biallelic pathological variants in the dopa decarboxylase (*DDC*) gene that encodes for the AADC enzyme.^1^^,^^2^ Deficiency of the AADC enzyme leads to an inability to synthesize dopamine and serotonin from their precursors, L-3,4-dihydroxyphenylalanine (L-DOPA) and 5-hydroxytryptophan (5-HTP).^3^ Without neuronal dopamine, patients suffer from movement disorders including hypokinesia, dystonia, and oculogyric crisis that result in motor dysfunction, along with behavioral problems, autonomic dysfunction, and developmental delay.^3^, ^4^, ^5^ Signs and symptoms of AADC deficiency can vary greatly. A recent survey revealed that most patients have a severe disability with no acquisition of head control, with some mild outliers and intermediate presentation.^6^ The prevalence of AADC deficiency is higher in the Chinese population due to the presence of a founder splice variant, c.714+4A>T (IVS6+4A>T), which causes an insertion of 37 nt from intron 6 into the *DDC* mRNA.^7^ This variant, which is associated with a severe disease phenotype, should still allow for a small amount of normally spliced product so affected homozygotes can survive.^3^^,^^8^ A diagnosis of AADC deficiency typically relies on detection of low cerebrospinal fluid (CSF) dopamine and serotonin metabolites, homovanillic acid (HVA) and 5-hydroxyindoleacetic acid (5-HIAA),^2^^,^^3^ respectively, although molecular diagnosis has become a standard practice. Moreover, we have demonstrated that 3-O-methyldopa (3-OMD) from a dried blood spot is a convenient biomarker for the diagnosis of AADC deficiency.^9^ Newborn screening using 3-OMD concentration in dried blood spots has also been developed.^10^, ^11^, ^12^

Currently approved treatment options are limited to attempts to increase monoamine neurotransmitter production; decrease their catabolism through the inhibition of monoamine oxidase (MAO); or address symptomatic concerns such as nasal congestion, difficulties with sleep, and irritability.^1^^,^^3^ These therapies provide variable results and do not treat the underlying cause of disease, which is related to the insufficiency or absence of AADC activity. Studies in adults with Parkinson disease have shown that the intraputaminal infusion of the adeno-associated virus type 2 (AAV2) vector-mediated delivery of the human AADC gene increases AADC enzymatic activity, with good safety and tolerability profiles.^13^, ^14^, ^15^ Because the main symptoms of AADC deficiency include reduced brain dopamine levels and motor function impairments, this disease would be a promising candidate for a gene therapy approach similar to that used in Parkinson disease.

Recombinant AAV2 vector containing the human AADC gene (rAAV2-hAADC, eladocagene exuparvovec), was developed as a sterile parenteral formulation gene therapy, containing the active biological substance recombinant and compendial excipients, delivered to cells within the putamen to drive production of the AADC enzyme. The first gene therapy trials showed that intraputaminal infusion of eladocagene exuparvovec is well tolerated and improves motor development in children with AADC deficiency.^16^, ^17^, ^18^ However, these earlier observations had limitations, such as the small population and lack of long-term follow-up; long-term follow-up is necessary to evaluate a therapy's efficacy, durability, and the risk of delayed adverse events over time.^19^

Herein, authors report the combined results of a long-term follow-up from the three eladocagene exuparvovec gene therapy trials. These analyses provide two important advancements in understanding the use of gene therapy in patients with AADC deficiency. The first is the ability to look at results in a total population of 26 patients with AADC deficiency who have completed 1-year evaluations, allowing for a better understanding of the potential relationship between patient characteristics and dosage on outcomes. The second is the evaluation of long-term efficacy and safety in 11 of the 26 patients, who have been followed for >5 years.

## Results

### Demographic information of the patients

A total of 26 patients equally distributed between male and female were enrolled in three consecutive trials (compassionate use, phase 1/2, and phase 2b) and have completed 1-year evaluations (Table 1). The three trials employed the same treatment protocol, with the exception of five patients in the phase 2b trial who received a 33% higher dosing (2.4 × 10^11^ versus 1.8 × 10^11^ vg). The enrollment criteria for the latter part of phase 2b trial slightly changed; only patients aged <6 years were eligible for a greater prospect of benefit. The trial enrolled patients of Chinese descent, with the exception of one patient, patient 307, who identified as Caucasian/Thai. The c.714+4A>T (IVS6+4A>T) splice variant represented 80.8% of all mutated *DDC* alleles (Table 1). Patient CU-02 was lost to follow-up after the 1-year evaluation and died 5 years after gene therapy, likely due to aspiration. Patient 1007 died of influenza B encephalitis before the 1-year evaluation; the patient's 9-month evaluation data were used as the 1-year data for statistical analyses. Patient CU-07 experienced periods of vomiting and diarrhea 1 month after gene therapy that resulted in hypovolemic shock, likely because of his autonomic system dysfunction; this shock was unrelated to treatment but caused hypoxemic encephalopathy. This patient's data were used for safety analysis only.^20^ As a whole, the 26 patients were surgically treated at a mean age of 4.1 ± 2.2 years (1.7–8.5 years), followed for a mean 5.4 ± 2.6 years (2.0–10.2 years), and the mean age in mid-2020 was 9.5 ± 4.0 years (4.2–16.6 years).

**Table 1:** Combined demographics and baseline characteristics

Patient no.	Sex	Current age^a^ (y)	Age at GT (y)	Time after GT (y)	Variant 1	Variant 2
CU-01	F	14.4	4.3	10.2	c.714+4A>T^b^	c.714+4A>T
CU-02^c^	M	9.6	4.5	5.2	c.714+4A>T	c.714+4A>T
CU-03	F	14.5	4.5	10.0	c.714+4A>T	c.714+4A>T
CU-04	F	15.5	6.2	9.3	c.714+4A>T	c.1297-1298 insA
CU-05	M	10.9	2.1	8.9	c.714+4A>T	c.714+4A>T
CU-06	F	11.3	2.7	8.7	c.714+4A>T	c.714+4A>T
CU-07	M	15.1	6.7	8.5	c.714+4A>T	c.714+4A>T
CU-08	F	16.6	8.3	8.4	c.714+4A>T	c.714+4A>T
1001	F	11.7	6.2	5.6	c.714+4A>T	c.714+4A>T
1002	M	13.2	7.7	5.5	c.714+4A>T	c.714+4A>T
1003	F	13.8	8.5	5.5	c.714+4A>T	c.714+4A>T
1004	M	7.8	2.5	5.4	c.714+4A>T	c.1058T>C (p.Leu353Pro)
1005	M	8.0	2.7	5.3	c.714+4A>T	c.714+4A>T
1006	F	11.7	6.5	5.2	c.714+4A>T	c.1297-1298 insA
1007^c^	M	3.6	2.7	1.0	c.714+4A>T	c.179T>C (p.Val60Ala)
1008	F	7.9	2.9	5.1	c.714+4A>T	c.286G>A (p.Gly96Arg)
1009	M	6.7	2.1	4.6	c.714+4A>T	c.714+4A>T
1010	F	6.2	1.7	4.5	c.714+4A>T	c.714+4A>T
301	M	9.3	5.8	3.6	c.714+4A>T	c.1234C>T (p.Arg412Tryp)
303	M	7.5	4.2	3.4	c.714+4A>T	c.304G>A (p.Gly102Ser)
304^d^	M	5.0	1.8	3.3	c.714+4A>T	c.714+4A>T
305	F	6.9	3.7	3.2	c.714+4A>T	c.714+4A>T
306^d^	M	4.7	1.7	3.0	c.714+4A>T	c.714+4A>T
307^d^	F	5.2	2.5	2.7	c.714+4A>T	c.179T>C(p.Val60Ala)
308^d^	M	4.5	2.0	2.5	c.714+4A>T	c.175G>A
309^d^	F	4.2	2.2	2.0	c.714+4A>T	c.1234C>T (p.Arg412Tryp)
Mean		9.5	4.1	5.4		
SD		4.0	2.2	2.6		

F, female; GT, gene therapy; M, male.

a Data cutoff, December 31, 2020.

b All c.714+4A>T was previously named IVS6+4A>T.

c At the last follow-up.

d Indicates high-dose group.

### Improvement in motor and cognitive function after gene therapy

**Figure 1:**
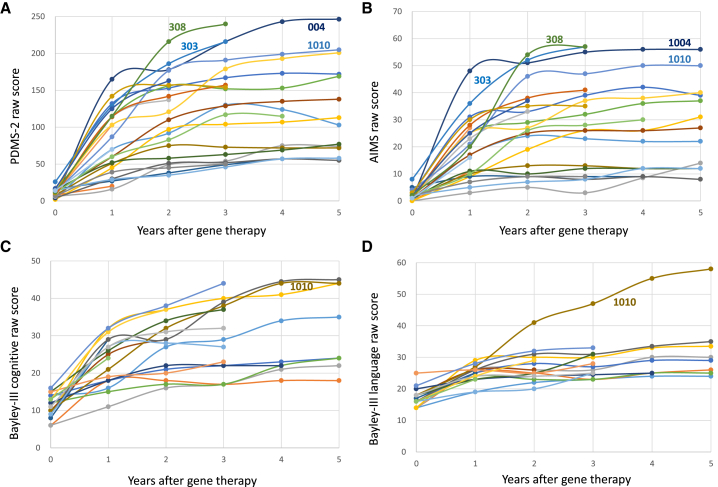
Improvements in developmental milestones after gene therapy Patients were evaluated and data were plotted according to years after gene therapy. (A) All patients exhibited rapid increases in PDMS-2 score after gene therapy. Three patients who could walk without assistance are marked with patient numbers 303, 308, and 1004. (B) AIMS score. (C) Cognitive score of Bayley-III for the phase 1/2 and phase 2b patients. (D) Language score of Bayley-III; patient 1010 exhibited an extraordinary score.

Peabody Developmental Motor Scales–Second Edition (PDMS-2) and Alberta Infant Motor Scale (AIMS) are universal tools to measure children's motor ability; AIMS focuses on gross motor skills, while PDMS-2 includes fine motor function. These two tests were used throughout the three studies. In the compassionate use study, Comprehensive Developmental Inventory for Infants and Toddlers (CDIIT) was used to measure cognitive function and language ability, while, in the two subsequent studies, the cognitive and language scales of the Bayley Scale of Infant and Toddler Development, Third Edition (Bayley-III) were employed. Before gene therapy (the baseline evaluation), patients had a very low mean PDMS-2 score of 10.4 ± 5.4 (n = 25). This score increased rapidly at 1 year (80.5 ± 43.4; n = 25), 2 years (114.5 ± 55.2; n = 22), and 5 years after gene therapy (116.1 ± 59.8; n = 11); the score at each time point was significantly higher compared with baseline (p < 0.01 for each) (Figure 1A). AIMS score increased from baseline (1.8 ± 1.8) at 1 year (18.8 ± 11.0), 2 years (26.9 ± 15.5), and 5 years (24.5 ± 15.0; each p < 0.001; Figure 1B) post treatment. CDIIT score in the compassionate use patients increased from baseline (19.9 ± 9.7; n = 7) at 1 year (45.4 ± 19.2; n = 7; p = 0.004), 2 years (65.8 ± 20.6; n = 6; p = 0.003), and 5 years (62.2 ± 23.0; n = 5; p = 0.002) following therapy. Bayley-III cognitive score in other patients increased from baseline (11.2 ± 3.0; n = 18) at 1 year (23.2 ± 6.4; n = 18; p < 0.001), 2 years (27.3 ± 7.4; n = 16; p < 0.001), and 5 years (27.8 ± 9.7; n = 6; p = 0.006; Figure 1C). Finally, improvements from baseline Bayley-III language (17.2 ± 2.8; n = 18) were noted at 1 year (24.6 ± 2.6; n = 18; p < 0.001), 2 years (26.9 ± 5.0; n = 16; p < 0.001), and 5 years (27.9 ± 3.6; n = 6; p = 0.007; Figure 1D) after treatment with eladocagene exuparvovec. Only a few patients tried Neupro (Rotigotine Transdermal System), which is a non-ergoline D3/D2/D1 dopamine agonist, 1 year after the surgery, but no treated patient used Neupro currently.

Three patients could walk freely without assistance at the time of this follow-up (patient 1004, 303, and 308, at 2.9, 2.4, and 2.2 years after gene therapy, respectively). Their highest PDMS-2 scores were >200 (Figure 1A; healthy 3-year-old children may have a score of 400). These three patients were treated at a young age (2.5, 4.2, and 2.0 years, respectively), and gained motor function rapidly during the first 2 to 3 years after gene therapy. Patient 1004 was able to run freely at the 5-year evaluation (Videos S1 and S2). Patient 1010 had the ability to talk 3.4 years after gene therapy; her language score reached 60 at the age of 5 years (Figure 1D; healthy 3-year-old children may have a score of 70). Her PDMS-2 (Figure 1A), AIMS (Figure 1B), and cognitive (Figure 1C) scores were also high, and she was treated at the youngest age among all, 1.7 years. Among these four patients (1004, 1010, 303, 308), only patient 308 received the high dose.

Video S1. Clips from the baseline motor evaluation of Patient 004, at the age of 2.5 years

Video S2. Clips from the 5-year evaluation of the same individual

### Objective evidence of efficacy

**Figure 2:**
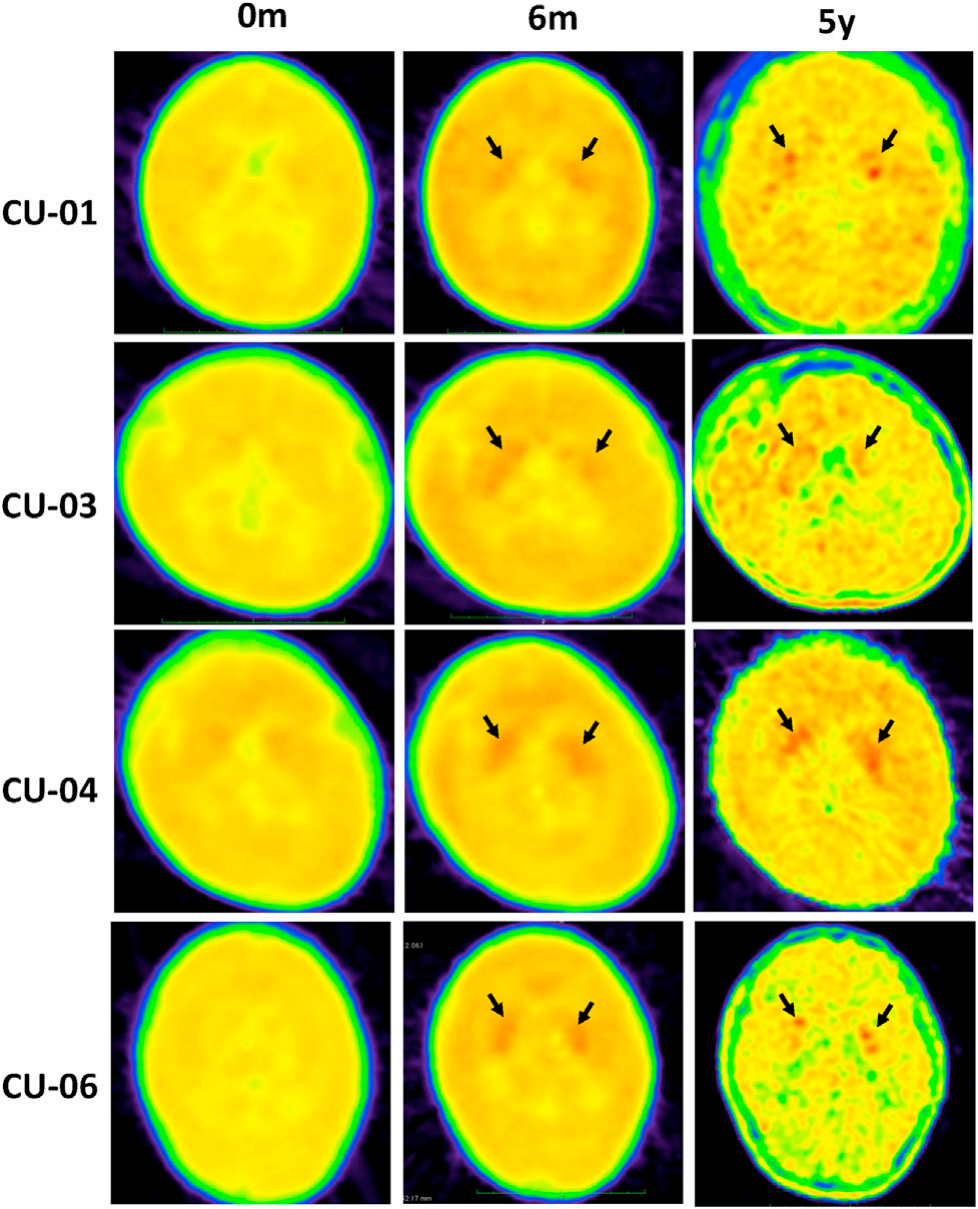
*De novo* dopamine production: visualized ^18^F-DOPA PET increases in four patients Each row shows ^18^F-DOPA PET scans of the putamen at baseline (0 months), 6 months (except for patient CU-06 at 12 months), and 5 years. Black arrows indicate the observed signal.

CSF analyses of HVA and 5-HIAA reflect the levels of dopamine and serotonin in the brain. Before gene therapy, patients had very low levels of HVA in the CSF (6.6 ± 11.2 nmol/L), and the levels increased to 30.2 ± 16.7 nmol/L 12 months after gene therapy (p < 0.001). CSF HIAA levels before (9.2 ± 14.5 nmol/L) and after (5.0 ± 10.1 nmol/L) gene therapy did not significantly differ (p = 0.33). Positron emission tomography (PET) analyses reflected AADC activity inside the putamen that L-6-[^18^F] fluoro-3, 4-dihydroxyphenylalanine (^18^F-DOPA) can be converted to ^18^F-dopamine and taken up by the nerve terminals in the putamen (Figure 2). At baseline, patients had a mean ^18^F-DOPA-specific uptake of 0.23 ± 0.14 (n = 24) that increased 12 months after gene therapy (0.48 ± 0.24; n = 24, p < 0.001), 2 years (0.55 ± 0.24; n = 15; p = 0.003), and 5 years (0.60 ± 0.20; n = 13; p < 0.001). PET data at 5 years demonstrated the durability of gene transduction effect and were consistent with the durability of motor milestone development.

### Growth, symptoms, and life quality analyses

**Figure 3:**
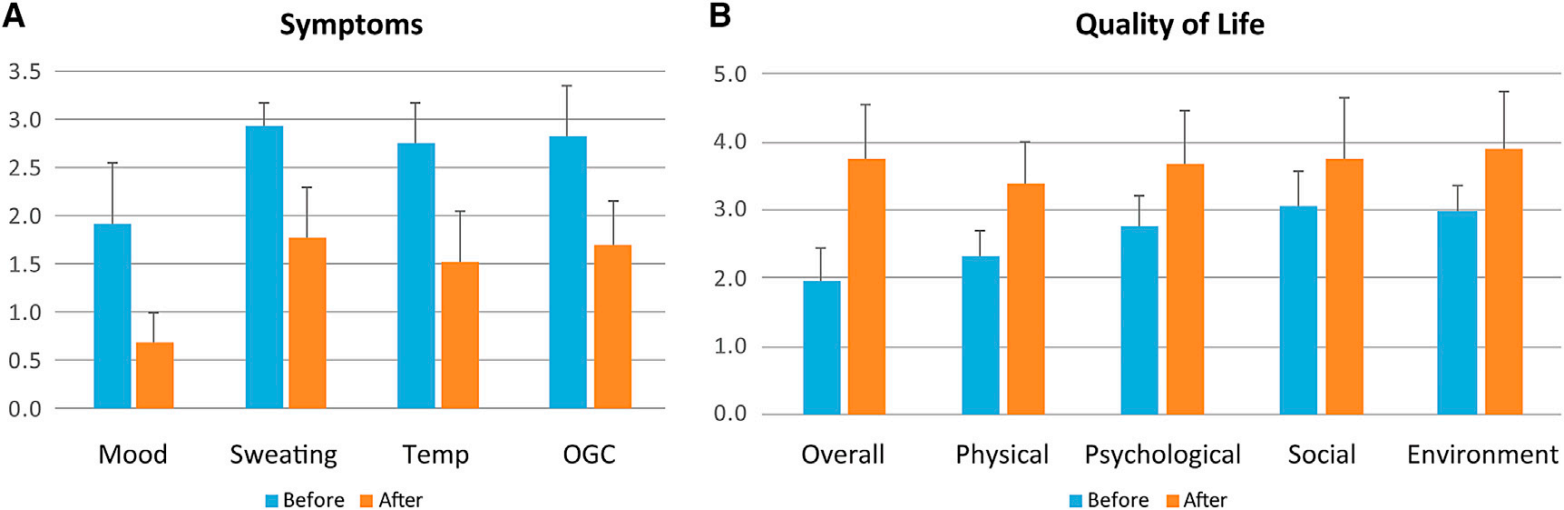
Improvements in symptoms of the patients and quality of life of the caregivers Mothers were asked to evaluate their own quality of life and the symptoms of the patients at the end of 2020 (after), and to recall the conditions before gene therapy (before). (A) Results of symptom severity score of the patients (higher score indicates more severe). (B) Results of WHOQOL-BREF Taiwan version (higher score indicates better quality) of the caregivers. Bars over the column indicate 1 standard deviation.

Patients with severe AADC deficiency usually stopped gaining weight after 1 year of age.^8^ In the current follow-up analyses, patients' body weight gain within 1 year before gene therapy was 9.4% ± 15.4% (n = 24). A spurt of weight gain occurred a few months after gene therapy, and weight gain in the year after gene therapy was 26.0% ± 17.5% (n = 24), significantly greater than at baseline (p = 0.001). Weight gain in the second year after gene therapy, 17.6% ± 13.7% (n = 21), was also elevated but was not statistically higher than at baseline (p = 0.065). While 21 of 26 patients had a body weight lower than the third percentile of normal at baseline; only 11 of the 23 patients with >1 year of follow-up had a body weight lower than the third percentile at the latest visit (p = 0.02, chi-square test). The change in body weight following treatment plotted alongside body weight growth curves for age- and gender-matched Taiwanese children without AADC deficiency is shown in Figure S1. At the end of the study period, questionnaires for retrospective assessment of caregivers' quality of life and patients' symptoms were sent to patients' mothers (n = 18), who lived in Taiwan and were considered main caretakers of the affected children; of the 18, 17 questionnaires were returned. The symptom severity questionnaires, with scores of 1 = normal, 2 = mild, and 3 = severe, revealed that the severity of proportion of bad mood, excessive sweating, temperature instability, and severity of oculogyric crises all decreased significantly (p < 0.001; Figure 3A). The World Health Organization Quality of Life (WHOQOL)-BREF (Taiwan version) is a widely used tool to measure quality of life. The results revealed that caregivers had improved quality of life after gene therapy in all five domains: overall (p < 0.001), physical health (p < 0.001), psychological (p < 0.001), social relationship (p = 0.006), and environment (p < 0.001; Figure 3B). Only three of the 28 questions in the questionnaire did not reach significant improvement: sex life (p = 0.069), support from friends (p = 0.096), and transport (p = 0.058).

### Factors correlating with response to gene therapy

**Figure 4:**
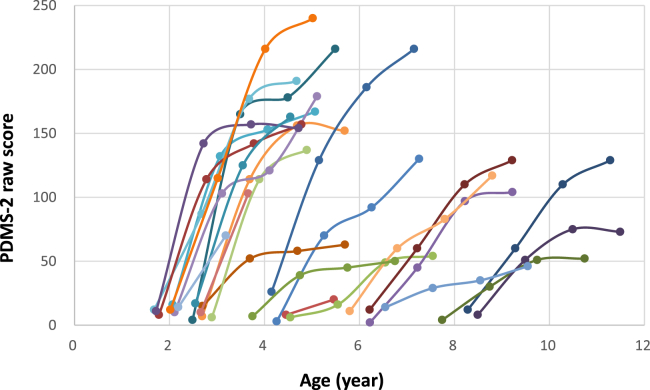
PDMS-2 score, by patient and chronological age PDMS-2 scores of individual patients (N = 26) at baseline and 1 year after gene therapy. Each line graph shows PDMS-2 total score in each patient. The first data point for each patient indicates baseline score at the time of eladocagene exuparvovec administration.

Age at time of treatment exhibited a significant correlation with the response to therapy as measured by 1-year (p < 0.001) and 2-year (p < 0.001) post-treatment PDMS-2 scale after gene therapy (Table S1). We plotted the age against PDMS-2 score for each patient over the follow-up periods in Figure 4, and we could clearly see that curves on the left side of the chart (the younger ones) rose more acutely than those on the right (the older ones). A strong correlation was observed between the post-treatment HVA level and PDMS-2 scores, suggesting that motor function improvements were contributed from dopamine production as enabled by the gene therapy product delivered (Table S1). Interestingly, a moderate correlation was also observed between the pre-treatment HVA level and post-treatment PDMS-2 score, even though all patients were severely affected before the treatment, as evident from the lack of correlation between pre- and post-treatment PDMS-2 scores (Table S1). There was no correlation between dosage and 1-year (p = 0.40) or 2-year (p = 0.09) post-treatment PDMS-2 scores (not adjusted for age). In our studies, patients were not randomized by age or dose, and in linear regression analyses of age at gene therapy, peak antibody titer, pre-treatment HVA, and dosage, only age was correlated to 1-year (p = 0.004) and 2-year (p = 0.008) post-treatment PDMS-2 score.

### Safety analysis

**Figure 5:**
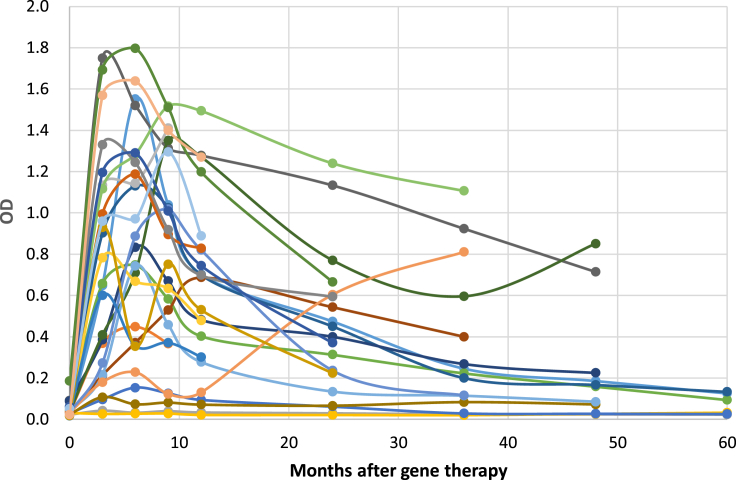
Antibody production against AAV2 vector at different time points after treatment The line graphs show the anti-AAV2 antibody serum levels in each patient (N = 26).

Most patients had a positive anti-AAV2 antibody response within the first year after eladocagene exuparvovec treatment. Titers increased rapidly after infusion and declined in the majority of patients beyond the 6-month point (Figure 5). Statistics show correlations between peak total antibody titer and 1-year (R = 0.46; p = 0.027) and 2-year (R = 0.54, p = 0.012) post-treatment PDMS-2 score, and between first-year antibody titer and 1-year (R = 0.43, p = 0.041) and 2-year (R = 0.58, p = 0.006) post-treatment PDMS-2 scores. Because immune responses to the vector capsid may compromise therapeutic expression of the transgene in systemic gene therapy^21^ but immune responses are not expected to affect localized brain gene therapy, these weak positive corrections should not be clinically relevant.

**Figure 6:**
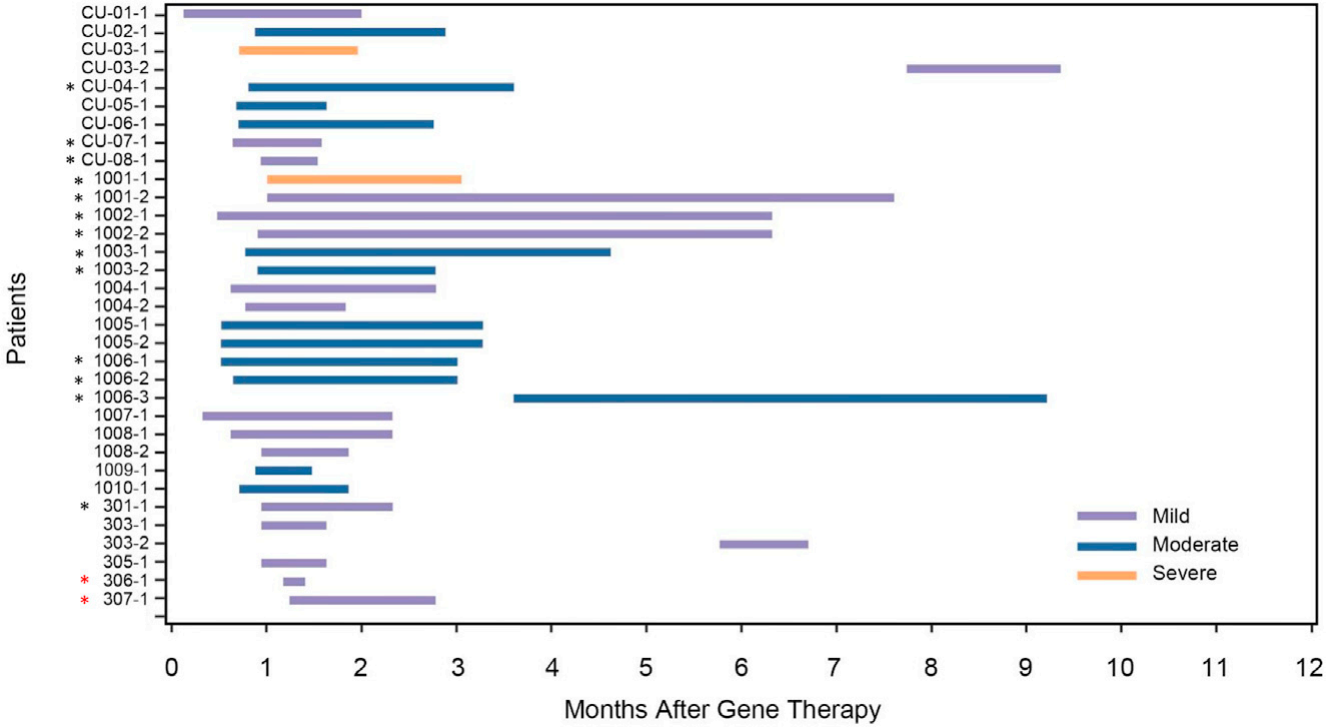
Dyskinesia events observed through 12 months The duration and severity of dyskinesia events (purple indicates mild; blue indicates moderate; orange indicates severe) were charted for each patient, as well as how many months after treatment they occurred. Overlapping events (e.g., generalized or orofacial dyskinesia) were reported in nine patients and are differentiated by Arabic numeral after case number. One event of dyskinesia (patient CU-03) that occurred at 43 months after treatment is not included in this graph. Black stars indicate events that occurred in patients who received gene therapy at an age >5 years. Red stars indicate events that occurred in patients who received a high dose.

Ten patients experienced adverse events potentially related to the surgical procedure, including CSF leakage, the majority of which occurred the day of or day after surgery, and all resolved (Table 2). To prevent leakage and stabilize the wound, the surgical procedure was modified to include a titanium mesh to cover the burr hole. All patients experienced at least one treatment-emergent adverse event (TEAE) during the study. The most commonly reported TEAEs were pyrexia and dyskinesia (Table 2). Two deaths were reported; one patient experienced encephalitis due to influenza B 11 months after study treatment and subsequently died, and the other patient died of likely aspiration 5 years after gene therapy. Overall, 24 patients reported a total of 35 dyskinesia events (Figure 6). Most dyskinesia events were mild or moderate in severity and occurred ≤3 months after eladocagene exuparvovec administration. Dyskinesia tended to be more severe and prolonged in patients who received gene therapy at an older age (>5 years; Figure 6, black stars), but was not related to dosage (high dose; Figure 6, red stars). These events likely resulted from dopamine receptor hypersensitivity and thus were related to eladocagene exuparvovec treatment. All events resolved within 10 months after treatment. Only one event of dyskinesia occurred >12 months after eladocagene exuparvovec treatment, at 43 months in patient CU-03; this patient responded partially to gene therapy, and this event was likely related to her underlying disease rather than gene therapy.

**Table 2:** Summary of adverse events related to surgery and TEAEs (n = 26)

	n (%)
Adverse event category	
Injury, poisoning, and procedural complications	
Endotracheal intubation complication	1 (3.8)
Skin injury	1 (3.8)
Subcutaneous hematoma	1 (3.8)
Transfusion reaction	1 (3.8)
Wound complication	1 (3.8)
Nervous system disorders	
CSF leakage	3 (11.5)
Vascular disorders	
Hypotension	6 (23.1)
TEAEs occurring in ≥50% of patients	
Pyrexia	25 (96.2)
Dyskinesia	24 (92.3)
Upper respiratory tract infection	18 (69.2)
Gastroenteritis	17 (65.4)
Pneumonia	17 (65.4)
Upper GI hemorrhage	15 (57.7)
Diarrhea	13 (50.0)

GI, gastrointestinal.

### Emerging longer follow-up data: patients from CU study

Patients in the CU study were followed for the longest period, 10 years for patient CU-01 and patient CU-03, 8 years for patient CU-04, 7 years for patient CU-05, and 6 years for patient CU-06, although a separate statistical analysis was not possible owing to the small sample size (Figure S2A). Patients CU-03, CU-04, and CU-06 were stable in PDMS-2 and AIMS scores. One patient had left knee growth plate injury by infection before gene therapy, resulting in an angled left lower leg, which interfered with the ability to stand and walk. The patient exhibited a gradual decline in motor scores 3 years after gene therapy. The patient underwent leg surgery to straighten the left leg at 7 years after gene therapy, and motor function was stabilized thereafter. Patient CU-05 exhibited a decline in PDMS-2 and AIMS scores after 5 years, although still markedly higher than pre-treatment baseline, so we performed a panel of examinations at 7 years after gene therapy. He had a homozygous c.714+4A>T genotype, similar to half of our patients, and his peak anti-AAV2 titer (optical density [OD], 0.153), at 6 months after gene transduction, was not high. This patient's magnetic resonance imaging (MRI) scan revealed only the cannula tract but no brain pathology or abnormal enhancement at the site of dosing, perhaps a feature of AAV2 uses in the studies (Figure S2B); CSF analysis revealed no increase in protein level, no increase in cell counts, and no decrease in glucose. The CSF HVA level (40 nmol/L) was similar to the level (37 nmol/L) at 6 months after gene therapy, and PET study revealed stable expression of AADC activity (Figure S2C), confirming sustained gene therapy product production. We then found that the patient quickly became dystonic when he underwent training or examination, which likely contributed to the decline of PDMS-2. We performed aquatic therapy to ameliorate the dystonic symptom.

## Discussion

Adeno-associated virus vectors are suitable for gene transduction in nondividing target cells because the vectors can persist as episomes in the nuclei.^22^ In animal studies, hemophilia A dog models were shown to have persistent AAV8-mediated transgene expression for ≤10 years, although vector genome integration in the hepatocytes was questioned.^23^^,^^24^ Persistent expression of dopamine-synthesizing enzymes, including AADC, has been shown 15 years after gene transfer in a primate model of Parkinson disease.^25^ Recently, long-term outcomes of AAV gene transfer in human trials have drawn intense attention.^23^ In ocular gene therapy trials, substantial improvements in visual acuity for ≥4 years were noted in patients receiving AAV gene transfer for correction of Leber congenital amaurosis.^26^ In gene therapy for hemophilia, the expressions of factor IX and factor VIII were observed for ≤3 years.^27^^,^^28^ The current study thus demonstrated the longest therapeutic effect of AAV-mediated gene therapy for 5 years, robustly proven by restoration of ^18^F-DOPA-specific uptake in the putamen, increase in CSF HVA concentration, and improvements in motor function.

We discovered two prognostic factors: age and pre-treatment HVA level. The increase in PDMS-2 total scores after eladocagene exuparvovec treatment had a negative correlation with age (Table S1), indicating that younger patients exhibited faster and greater improvements in PDMS-2 scores after gene therapy, a finding similar to previous publications.^16^^,^^18^ This is likely related to a greater degree of neuronal plasticity in younger patients. A positive correlation was also observed between PDMS-2 score and the pre-treatment HVA levels. This suggests that the presence of pre-treatment HVA may indicate a slight decrease in disease severity, and although not clinically recognizable, it may be associated with better treatment outcomes. However, the data are still limited and need to be explored further.

The safety of eladocagene exuparvovec treatment has been documented in previous studies.^16^^,^^17^ This report adds to the existing body of evidence on the eladocagene exuparvovec safety by further assessing the short- and long-term (≥5 years) tissue damage due to surgical intervention. MRI from a patient at 7 years after treatment showed evidence of the tracts from the operation, with no additional tissue damage. Dyskinesia usually appeared 4 weeks after treatment with eladocagene exuparvovec, peaked at 8 weeks, and subsided a few weeks thereafter. In view of the transient character of this symptom, dopamine receptor hypersensitivity after long-term dopamine deficiency is likely the cause. In several patients in whom functional motor movement cannot develop successfully, for example in patient CU-03, probably due to the lack of neuroplasticity, dyskinesia may persist.

There are limitations to this research. First, in view of the remarkable therapeutic effect and the post-gene delivery dyskinesia, we did not perform aggressive dose escalation. Second, we only injected to the putamen and not to the midbrain, where serotonin is produced, but most of our patients also experienced an improvement in mood after treatment. A recent study also demonstrated therapeutic effects of substantia nigra gene therapy in a smaller cohort of patients (n = 7) with AADC deficiency.^29^^,^^30^ However, the intraputaminal site of administration may be preferable because of its high safety profile and possible restoration of the prefrontal corticoputaminal network.^29^^,^^31^ Third, we did not correct systemic catecholamine deficiency, which may lead to hypoglycemia and hypotension, but these events are preventable. A systemic gene therapy, as we previously demonstrated in the AADC-deficiency mouse model,^7^ may be helpful to treat disease affecting the midbrain and the autonomic nervous system. Fourth, although we demonstrated that younger patients had a better outcome, we were not able to treat patients aged <1.5 years owing to the unstable skull structure of the partially fused anterior fontanel. Further, we did not measure specific immune responses to the transgene product because of negative findings in brain MRI and the difficulty of assessing the immune responses in the brain.

Overall, the current study demonstrated the safety and durable efficacy of intraputaminal infusion of eladocagene exuparvovec for AADC deficiency, which is the longest follow-up of AAV-mediated therapeutic effect, up to 9 years. To date, newborn screening of AADC deficiency has been feasible,^12^ and gene therapy immediately after newborn screening could potentially cure the disease. However, new technology like robotic brain surgery that can operate on a flexible skull will be necessary to enable the operation on infants.

## Materials and methods

### Study design and participants

All studies were approved by the appropriate institutional and/or national research ethics committee and have been performed in accordance with the ethical standards as described in the Declaration of Helsinki. Three single-arm, open-label clinical trials were included in this study. Patients needed a confirmed diagnosis of AADC deficiency by fulfilling all the following criteria: decreased levels of CSF HVA and 5-HIAA, elevation of blood or CSF 3-OMD levels, presence of ≥one pathogenic *DDC* variant, and classical symptoms of AADC deficiency. At the time of the surgery, patients were aged ≥2 years or, if younger, had skull bones suitable for the surgery (closed anterior fontanelle).^32^ AAV2-hAADC (eladocagene exuparvovec) was used in this study and consisted of a cytomegalovirus immediate-early promoter followed by the first intron of human growth hormone, human AADC cDNA, and the simian virus 40 polyadenylation signal sequence. All patients underwent a stereotactic surgery for infusion at two target points per putamen at a rate of 3 μL/min. Noninvasive MRI was used to locate the infusion site and to monitor post-surgical complications. The compassionate use study (AADC-CU, patients CU-01–CU-08, eight patients) and phase 1/2 (AADC-010; NCT01395641, patients 1001–1010, 10 patients) trial used the same dosing, where patients received a total dose of 1.81 × 10^11^ vg of eladocagene exuparvovec. The phase 2b trial (AADC-011; NCT02926066, patients 301–309, eight patients) used the same dosing, but then excluded patients aged >6 years and shifted to 33% higher dosages of 2.4 × 10^11^ vg (n = 5). Drugs treating AADC deficiency, including dopamine agonists, MAO inhibitors, and anti-cholinergic drugs, could not be used 1–12 months after the surgery, except that risperidone (a dopamine D_2_ receptor antagonist) could be used for post-gene-delivery dyskinesia. Short-term outcomes of four patients from the compassionate use study^17^ and 10 patients from phase 1/2 trial^16^ have been reported.

### Clinical trial information

A phase I/II clinical trial for treatment of aromatic L-amino acid decarboxylase (AADC) deficiency using AAV2-hAADC (AADC): Clinicaltrials.gov, https://clinicaltrials.gov/ct2/show/NCT01395641; AADC-010; NCT01395641.

A clinical trial for treatment of aromatic L-amino acid decarboxylase (AADC) deficiency using AAV2-hAADC - an expansion: Clinicaltrials.gov, https://clinicaltrials.gov/ct2/show/NCT02926066 AADC-011; NCT02926066; National Taiwan University Hospital Taipei, Taiwan.

### Outcome measurements

The key efficacy endpoint for motor development was the PDMS-2 scores.^33^ PDMS-2 raw scores, rather than transformed scores, were used for statistics. Additional motor efficacy endpoints were raw scores for the AIMS.^34^ The CDIIT, used in the compassionate use study, is a test of five areas used to evaluate the development of infants and toddlers in the domains of cognition, language, motor skills, social skills, and self-care skills.^35^ Bayley-III^36^ was used in the phase 1/2 and phase 2b trials. Measurements were performed every 3 months in the first year after gene therapy, and every 6 months to 1 year thereafter.

### Pharmacodynamic evidence of dopamine production

Evidence of *de novo* dopamine production were derived from PET imaging with ^18^F-DOPA tracer, as well as CSF concentrations of dopamine and serotonin metabolites HVA and 5-HIAA. PET studies were performed before and 1 year after gene therapy and 5 years after gene therapy if feasible. CSF sampling was performed before and 1 year after gene therapy. In the compassionate use study, CSF and PET studies were performed both 6 months and 1 year after gene therapy. Levels of ^18^F-DOPA were expressed as the standardized uptake value (SUV) using the open-source, free medical image viewer Horos. All values used are the maximal SUV (SUV_max_). The left and right putamen SUV_max_, relative to the control region of the brain in the occipital lobe where no AADC activity was expected, were subsequently averaged to provide a single putamen SUV_max_ measurement. CSF samples were obtained by lumbar puncture.

### Immunogenicity analysis

Immunogenicity was monitored at months 3, 6, 9, and 12, and then annually by ELISA.^32^ OD values were used in the statistical analysis. Because pre-existing anti-AAV2 antibody is not likely to affect the efficacy or safety of current localized brain injection, only patients with high anti-AAV2 titers would be excluded by the current study. A neutralizing antibody assay was used in the compassionate use study, but these samples were reassayed by the ELISA assay so data can be pooled together for analysis.

### Questionnaires

The in-house symptom questionnaires (in Chinese) included four severity scores: proportion of bad mood, excessive sweating, temperature instability, and severity of oculogyric crises. The scores were 1 = normal, 2 = mild, and 3 = severe. The WHOQOL-BREF Taiwan version contains 28 questions covering five domains: overall (two questions), physical health (seven questions), psychological (six questions), social relationship (four questions), and environment (nine questions).^37^ The scores ranged from 1 to 5, where 1 represented the lowest score and 5 the best. The two questionnaires were sent to patients' mothers (n = 18) who lived in Taiwan and were the main caregivers of the affected child. In Taiwan, mothers are the usual caretakers of children. If the mothers cannot play the role, either the fathers, grandparents, other relatives, or a hired caretaker will take over the job. Therefore, we excluded patients for whom mothers were not the caretakers. The mothers were asked to evaluate the current symptoms of the child and the mother's own quality of life and were also asked to recall the conditions before gene therapy.

### Statistical analyses

The Pearson coefficient was used to evaluate the correlation between different parameters, and t tests were used for comparisons. For CSF biochemical analysis, values that were below the limit of detection were assessed as 0. Missing data between two timepoints were imputed geometrically. Linear regression analyses were conducted by analysis of variance using the SPSS software (version 16.0).

## Data availability

Data collected from this study, including deidentified individual patient narratives, will be made available after publication of this article upon reasonable request to the corresponding author.

## Supplemental information

Document S1. Figures S1 and S3 and Table S1

Document S2. Article plus supplemental information
