# Supplementary material for: Long-term efficacy and safety of eladocagene exuparvovec in patients with AADC deficiency
Source: Mol Ther. 2021 Nov 8;30(2):509–18. doi: 10.1016/j.ymthe.2021.11.005 (PMC8822132; doi:10.1016/j.ymthe.2021.11.005)
Supplement: Document S1. Figures S1 and S3 and Table S1 [file mmc1.pdf]

YMTHE, Volume *30*

Supplemental Information

Long-term efficacy and safety of eladocagene exuparvovec in patients with AADC deficiency

Chun-Hwei Tai, Ni-Chung Lee, Yin-Hsiu Chien, Barry J. Byrne, Shin-Ichi Muramatsu, Sheng-Hong Tseng, and Wuh-Liang Hwu

Table S1. Correlations Between Variables and Response to Eladocagene Exuparvovec

Predicting Factor, Pearson Coefficient	First-Year PDMS-2 Score	Second-Year PDMS-2 Score
Age at time of treatment, y	–0.698 (*P*<0.001)	–0.686 (*P*<0.001)
Pre-treatment CSF HVA	0.65 (*P*=0.001)	0.46 (*P*=0.04)
Post-treatment CSF HVA[a]	0.84 (*P*<0.001)	0.89 (*P*<0.001)
Pre-treatment PDMS-2 Score	0.21 (*P*=0.34)	0.25 (*P*=0.29)

CSF, cerebrospinal fluid; HVA, homovanillic acid; PDMS-2, Peabody Developmental Motor Scales–Second Edition.
[a]Post-treatment HVA measured at 1 year after therapy.

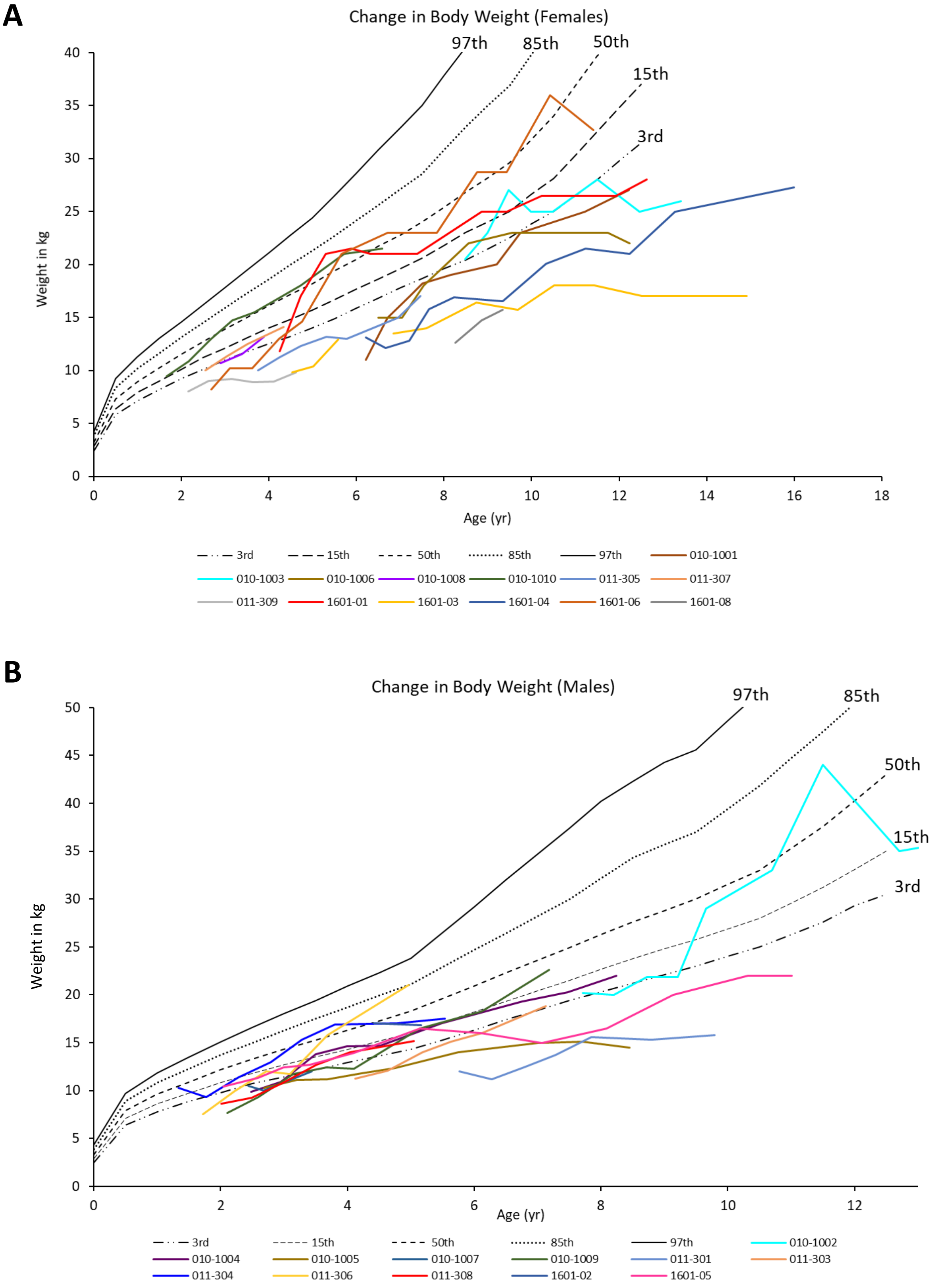

Figure S1. Change in body weight following treatment with eladocagene exuparvovec. (A) Change in body weight for 13 female patients ≤9 years after gene therapy plotted against patient age at time of measurement. **(B)** Change in body weight for 12 male patients ≤9 years after gene therapy plotted against age at time of measurement. Black solid and dashed lines indicate body weight growth curve percentiles for age- and gender-matched Taiwanese children without AADC deficiency.

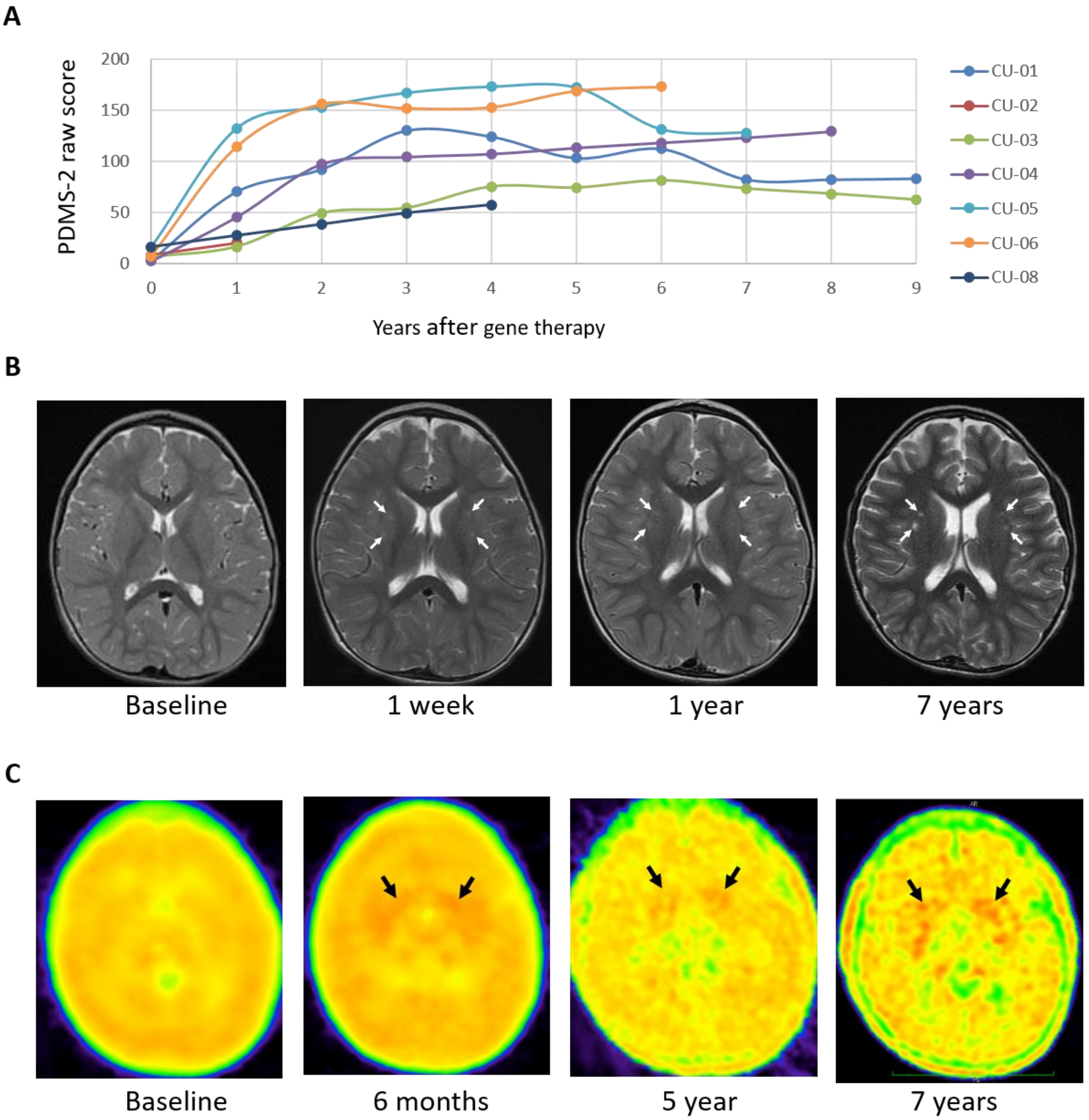

Figure S2. Efficacy and safety of eladocagene exuparvovec in the compassionate use. (A) PDMS-2 score of the 7 patients ≤9 years after gene therapy. **(B)** Horizontal T2-weighted MRI images of CU-05 at baseline, 1 week, 1 year, and 7 years after gene therapy. Signals caused by injection in dorsal putamen are marked by arrows. **(C)** ^{18}F-DOPA PET scans of the putamen at baseline, 6 months, 5 years, and 7 years after gene therapy. Black arrows indicate the observed signal. ^{18}F-DOPA, L-6-[^{18}F] fluoro-3, 4-dihydroxyphenylalanine; MRI, magnetic resonance imaging; PDMS-2, Peabody Developmental Motor Scales, Second Edition; PET, positron emission tomography.

Video S1. Clips from the baseline motor evaluation of Patient 004, at the age of 2.5 years.

Video S2. Clips from the 5-year evaluation of the same individual.
